# Concurrent Large Pericardial Cyst with Giant Thoracic Aortic Aneurysm—Presentation and Natural Evolution

**DOI:** 10.5152/eurasianjmed.2021.20172

**Published:** 2021-10

**Authors:** Jennie Han, Ekene Kenneth Okonkwo, Nadeem Attar

**Affiliations:** Department of Cardiology, Royal Lancaster Infirmary, Lancaster, United Kingdom

**Keywords:** Pericardial cyst, thoracic aortic aneurysm, aortic root dilation, computerised tomography of the thorax

## Abstract

We report the first case of a patient with large pericardial cyst occurring concurrently with a very large thoracic aortic aneurysm. There have been no patients reported in the literature with this constellation of syndromes. Additionally, this case was followed through a period of 4 years, enabling the natural evolution of both pathologies to be visualised.

## Case Report

A 76-year-old female presented to the Emergency Department in December 2019 with a 1-day history of chest and epigastric pain which became more severe during a routine dental check-up. She was an ex-smoker and had known hypertension, peripheral artery disease, and previous non-ST elevation MI.

**Figure 1.**
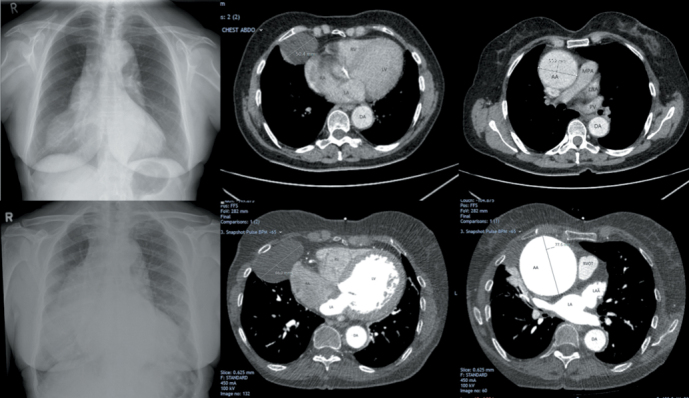
Chest X-ray and CT findings over the course of over 4 years. (A) *CXR March 2015, (B) CT showing pericardial cyst measuring 5 cm along the right heart border,* (C) CT showing ascending aortic aneurysm measuring 5.5 cm at the aortic root*, (D) CXR December 2019, (E) Cardiac gated CT showing pericardial cyst measuring 6.6 cm along the right heart border, and (F) cardiac gated CT showing giant ascending aortic aneurysm measuring 7.7 cm. Abbreviations: AA, ascending aorta; DA, descending aorta; LA, left atrium; LAA, left atrial appendage; LV, left ventricle; MPA, main pulmonary artery; PC, pericardial cyst; PV, pulmonary vein; RA, right atrium; RV, right ventricle; RVOT, right ventricle outflow tract*

Previous chest X-ray in March 2015 had shown a widened mediastinum with localised well-demarcated soft tissue opacity over the right heart border, suggestive of aortic dilatation and pericardial cyst, respectively (Figure 1a). Previous contrast enhanced computerised tomography (CT) thorax at the time showed a 5 cm pericardial cyst along the right heart border (Figure 1b) and a 5.5 cm ascending aortic aneurysm at the aortic root (Figure 1c).

This admission, chest X-ray showed increased soft tissue opacity and increased mediastinal widening (Figure 1d). Cardiac gated CT showed evolution of the pericardial cyst measuring 6.6 cm (Figure 1e) and giant ascending aortic aneurysm measuring 7.7 cm (Figure 1f). This also demonstrated moderate to severe left circumflex artery stenosis. Transthoracic echocardiogram revealed severe aortic regurgitation and moderate aortic stenosis.

She underwent urgent repair of ascending aorta and hemiarch, aortic valve replacement using a size 21 mm perimount valve, along with vein graft to left circumflex artery. In cardiac intensive care unit, she made a slow recovery, was successfully weaned off the ventilator, and remains well at follow up.

## Discussion

Pericardial cysts are rare benign intrathoracic lesions, with an incidence of 1 in 100,000.^1^ The diameter of most pericardial cysts is usually between 1 and 5 cm. Large pericardial cysts are rare, with only data from 12 cases available from 2000 to 2014 in PubMed. Most are asymptomatic and are found incidentally on radiological imaging. However, they can be associated with compression of adjacent structures, leading to chest pain and breathlessness, and more serious complications such as cardiac tamponade and sudden death.^2^ The pericardial cyst in our patient appears to be congenital and grew in size over years.

Giant thoracic aortic aneurysms are less benign, often from cystic degeneration of the media, leading to weakening of the vessel wall, aortic dilation and aneurysm formation.^3^ In total, 60% of thoracic aortic aneurysms involve annuloaortic ectasia of the aortic root or the ascending aorta, such as in this case. The giant aneurysm in our patient was due to hypertension along with atherosclerosis; there was no evidence of syphilis, connective tissue disorder or other predisposing factors.

Thoracic aneurysms are frequently asymptomatic and discovered incidentally on imaging. However, they can present with mass effects, aortic regurgitation if at the aortic root, and lead to life-threatening complications such as dissection or rupture. Aneurysms greater than 6 cm in diameter have a rupture or dissection rate of 6.9% per year and a death rate of 11.8%.^4^ The risk of rupture is exponentially linked to diameter. Generally, the literature supports considering intervention in aneurysms with a diameter of 4-5.5 cm.^5^ Due to this, our patient underwent urgent elective cardiothoracic repair of the aortic root, and she was fortunate not to have rupture or dissection at this massive size.

## References

[1] Patel J Park C Michaels J Rosen S Kort S. Pericardial cyst: Case reports and a literature review. Echocardiography. 2004;21:269-272. 10.1111/j.0742-2822.2004.03097.x 15053790

[2] Kar SK Ganguly T. Current concepts of diagnosis and management of pericardial cysts. Indian Heart J. 2017;69(3):364-370. 10.1016/j.ihj.2017.02.021 28648435 PMC5485391

[3] Isselbacher EM. Thoracic and Abdominal Aortic Aneurysms. Circulation. 2005;111(6):816-828. 10.1161/01.CIR.0000154569.08857.7A 15710776

[4] Davies RR Goldstein LJ Coady MA, . Yearly rupture or dissection rates for thoracic aortic aneurysms: Simple prediction based on size. Ann Thorac Surg. 2002;73(1):17-27. 10.1016/S0003-4975(01)03236-2 11834007

[5] Hiratzka LF Bakris GL Beckman JA, . 2010 ACCF/AHA/AATS/ACR/ASA/SCA/SCAI/SIR/STS/SVM guidelines for the diagnosis and management of patients with thoracic aortic disease: A report of the American College of Cardiology Foundation/American Heart Association Task Force on Practice Guidelines, American Association for Thoracic Surgery, American College of Radiology, American Stroke Association, Society of Cardiovascular Anesthesiologists, Society for Cardiovascular Angiography and Interventions, Society of Interventional Radiology, Society of Thoracic Surgeons, and Society for Vascular Medicine. Circulation. 2010;121(13):e266-e369 10.1161/CIR.0b013e3181d4739e. 20233780

